# Fuziline alleviates isoproterenol‐induced myocardial injury by inhibiting ROS‐triggered endoplasmic reticulum stress via PERK/eIF2α/ATF4/Chop pathway

**DOI:** 10.1111/jcmm.14803

**Published:** 2019-12-07

**Authors:** Cai‐lian Fan, Zhi‐hong Yao, Meng‐nan Ye, Lei‐lei Fu, Guo‐nian Zhu, Yi Dai, Xin‐sheng Yao

**Affiliations:** ^1^ College of Traditional Chinese Materia Medica Shenyang Pharmaceutical University Shenyang China; ^2^ College of Pharmacy and International Cooperative Laboratory of Traditional Chinese Medicine Modernization and Innovative Drug Development of Chinese Ministry of Education Jinan University Guangzhou China; ^3^ School of Life Science and Engineering Southwest Jiaotong University Chengdu China; ^4^ Research Core Facility West China Hospital Sichuan University Chengdu China

**Keywords:** apoptosis, ER stress, fuziline, isoproterenol, ROS

## Abstract

Fuziline, an aminoalcohol‐diterpenoid alkaloid derived from *Aconiti lateralis* radix preparata, has been reported to have a cardioprotective activity in vitro. However, the potential mechanism of fuziline on myocardial protection remains unknown. In this study, we aimed to explore the efficacy and mechanism of fuziline on isoproterenol (ISO)‐induced myocardial injury in vitro and in vivo. As a result, fuziline effectively increased cell viability and alleviated ISO‐induced apoptosis. Meanwhile, fuziline significantly decreased the production of ROS, maintained mitochondrial membrane potential (MMP) and blocked the release of cytochrome C, suggesting that fuziline could play the cardioprotective role through restoring the mitochondrial function. Fuziline also could suppress ISO‐induced endoplasmic reticulum (ER) stress via the PERK/eIF2α/ATF4/Chop pathway. In addition, using ROS scavenger NAC could decrease ISO‐induced apoptosis and block ISO‐induced ER stress, while PERK inhibitor GSK2606414 did not reduce the production of ROS, indicating that excess production of ROS induced by ISO triggered ER stress. And fuziline protected against ISO‐induced myocardial injury by inhibiting ROS‐triggered ER stress. Furthermore, fuziline effectively improved cardiac function on ISO‐induced myocardial injury in rats. Western blot analysis also showed that fuziline reduced ER stress‐induced apoptosis in vivo. Above these results demonstrated that fuziline could reduce ISO‐induced myocardial injury in vitro and in vivo by inhibiting ROS‐triggered ER stress via the PERK/eIF2α/ATF4/Chop pathway.

## 1 INTRODUCTION

Isoproterenol (ISO), a synthetic β‐adrenergic agonist, could induce myocardial apoptosis, cardiac hypertrophy and abnormality of diastolic and systolic functions at a certain dosage.^1^, ^2^ As the pathophysiological changes induced by ISO can mimic the state of pathological changes in cardiac tissue in the progress of human heart failure, the model of ISO‐induced myocardial injury has been widely used in exploring the cardioprotective effect of many drugs on cardiac function.^3^

Apoptosis is an evolutionarily conserved cell death pathway that plays an important role in regulating the homeostasis in multi‐cellular organism.^4^ Data of myocardial specimens from patients with severe myocardial injury have confirmed that the apoptotic index of myocardium was as high as 35.5% compared with 0.2%‐0.4% in the normal people.^5^ Therefore, reducing cardiomyocyte apoptosis is one of the important strategies to alleviate cardiac dysfunction and myocardial injury. Cardiomyocyte apoptosis could be caused by many factors such as oxidative stress, overload, inflammation and so on.^4^ There are two main pathways of apoptosis in eukaryotes: one is to activate the apoptotic enzyme in cells by extracellular signal, that is extrinsic pathway; the other is to activate apoptosis by releasing apoptotic enzyme activator from mitochondria, that is intrinsic pathway.^6^ Studies have proved that the intrinsic apoptotic pathway predominates under various stressors in heart.^6^, ^7^ The intrinsic apoptotic pathway is controlled by the release of pro‐apoptotic factors from mitochondria.^8^ Mitochondria are the metabolic centre of cell energy supply. Various stressors could lead to mitochondrial damage including structural damage, mitochondrial DNA mutation, ATP synthesis limitation, calcium homeostasis imbalance, accumulation of ROS and metabolic dysfunction.^9^, ^10^ Among these, excessive accumulation of ROS could cause severe oxidative stress injury directly leading to cell death. In addition, overaccumulation of ROS could increase ER stress to further aggravate cell death.^11^ A series of studies have shown that apoptosis in myocardial injury is also associated with endoplasmic reticulum (ER) stress injury. 11 ER stress is characterized by incorrect folding and aggregation of unfolded protein response (UPR) in endoplasmic reticulum lumen leading to the disorder of calcium balance, which could brake intracellular homeostasis to induce cell death.^12^ When the heart is stimulated by various stresses, cells will initiate ER‐related signalling pathways to degrade misfolded proteins quickly.^12^ Therefore, the appropriative level of ER stress could protect myocardial cells from injury. However, when persistent or severe injuries happened, ER stress would fail to release and endoplasmic reticulum homeostasis could not be reconstructed, resulting in the activation of apoptosis in UPR‐dependent or non‐dependent manner in cells.^13^, ^14^ Hence, inhibiting ER stress‐induced apoptosis maybe an effective way to alleviate myocardial injury.

Fuziline, a diterpenoid alkaloid, is mainly isolated from the Chinese herbal medicine *Aconiti lateralis* radix preparata. Previous study has found that fuziline could obviously improve the survival ratio of myocardial cells in the model of pentobarbital sodium‐induced myocardial injury in vitro*.* 15 However, the potential mechanisms of fuziline on myocardial protection have not been investigated. Therefore, we tried to use the models of ISO‐induced myocardial injury in vitro and in vivo to investigate whether fuziline exerts the cardioprotective effect by alleviating ISO‐induced oxidative and ER stress injury.

## 2 MATERIALS AND METHODS

### 2.1 Reagents and antibodies

Fuziline (CHB171026, MF: C24H39O7N, MW: 453.58, purity: 99.3%) was obtained from Chengdu Chroma‐Biotechnology Co., Ltd. (China) and prepared by dissolving in dimethyl sulfoxide as a 50 mmol/L stock solution. 3‐(4, 5‐dimetrylthiazol‐2‐yl)‐2, 5‐diphenyltetrazolium bromide (MTT) and ISO were purchased from Sigma‐Aldrich LLC (USA). 5,5′,6,6′‐tetrachloro‐1,1′,3,3′‐tetraethyl‐imidacarbocyanine iodide (JC‐1), 2′,7′‐dichlorodihydrofluorescein diacetate (DCFH‐DA) and MitoSOX were obtained from Beyotime Biotechnology Co., Ltd. (China). Annexin‐V‐FLUOS Staining Kit and Tunel Kit were purchased from Roche Co., Ltd. (USA). N‐acetylcysteine (NAC) and GSK2606414 were purchased from MedChemExpress Co., Ltd. (China). Antibodies against caspase‐3, p‐PERK, PERK, p‐eIF2α, eIF2α, GRP78, Chop, ATF4 and cytochrome C were obtained from Cell Signaling Technology Co., Ltd. (USA). Bcl‐2, Bax and GAPDH were purchased from Servicebio (China).

### 2.2 Cell culture and establishment of ISO‐induced myocardial injury model

H9c2 cells (rat embryonic ventricular myocytes) were purchased from the Shanghai Institute of Biochemistry and Cell Biology. High‐glucose DMEM added with 10% FBS, 100 μg/mL streptomycin and 100 U/mL penicillin was used to culture cells, and H9c2 cells were placed in an incubator with 37°C and 5% CO_2_. The medium was changed 2‐3 times per week.

The model of ISO‐induced myocardial injury in vitro was built as follows: when H9c2 cells have grown to a density of 80%, the medium was replaced by 80 μM ISO dissolved into high‐glucose DMEM with 1% FBS. Then, cells were incubated under the normoxic condition with air/CO_2_ (95:5%) at 37°C for 48 hours.

### 2.3 Cell viability assay

H9c2 cells (6 × 10^3^) were dispensed in 96‐well plates for 24 hours and then addressed with ISO and different concentrations of fuziline for the indicated time. Then, the DMEM was removed and 100 μL MTT solution (0.1 mg/well) was added, and H9c2 cells were incubated for 3 hours. After that, the supernatant was removed and the formazan crystals were dissolved in 150 μL DMSO. Then, the absorbance of each sample was detected at 490 nm by a microplate reader (Spectrafluor; TECAN, Sunrise, Austria). The percentage of cell viability was calculated in our study as follows: cell viability (%) = (A490, sample – A490, blank)/ (A490, control – A490, blank) × 100%.

### 2.4 Determination of cellular apoptosis

Apoptosis was measured by the Annexin‐V‐FLUOS Staining Kit according to the method of Piekarska et al.^16^ The apoptotic cells of each group were quantified through a flow cytometer (Beckman Coulter). The apoptosis rate was reflected via the ratio of Annexin‐V‐positive/PI‐positive cells to total cells.

### 2.5 Detection of mitochondrial membrane potential (MMP)

The change of MMP was detected by JC‐1 Assay kit under the guideline of the manufacturer's instructions. Briefly, H9c2 cells were distributed in six‐well plates at a density of 1 × 10^5^/well for 24 hours. Then, ISO and different concentrations of fuziline were added for 48 hours. H9c2 cells were harvested and washed third times with PBS and then resuspended in 1.5‐mL EP tubes with 1 mL culture medium. After that, 100 μL JC‐1 staining solution was added into tubes, and H9c2 cells were incubated for 15 minutes at 37°C in a CO_2_ incubator. After that, the samples were analysed by a flow cytometer (Beckman Coulter)*.*

### 2.6 Determination of intracellular ROS

The cellular ROS level was determined using ROS detection kits. In brief, following ISO and fuziline treatment, H9c2 cells were harvested and washed third with 1 × washing buffer and then resuspended in 0.5 mL serum‐free DMEM. The cell suspension was stained with 0.5 μL 100 mmol/L DCFDA for 30 minutes in the dark. The fluorescence intensity of DCFDA was quantified using a flow cytometer (Beckman Coulter).

### 2.7 Experimental animals and animal models of ISO‐induced myocardial injury

Male Sprague Dawley (SD) rats were provided by Hua Fukang Biotechnology Company (Beijing, China). The rats (weight 150‐200 g) were housed with five each in sanitized polypropylene cages at room temperature for 12‐hour light/dark cycle. The experiments were monitored by Laboratory Animal Ethics Committee of West China Hospital, Sichuan University.

ISO was injected intraperitoneally for 7 days at a dose of 5 mg/kg body weight to induce myocardial injury in rats. 17 LVEF < 50% by echocardiography was defined as heart failure. The control group (n = 8) was fed a dose of saline (6 mL/kg/day) for 4 weeks, and rats that were identified to be in a state of heart failure were randomly divided into three groups: ISO group (n = 8), fuziline‐treated group (3 mg/kg, intragastric administration, every day, n = 8) and metoprolol group (10 mg/kg, intragastric administration, every day, n = 8). After administration for 4 weeks, the rats were anaesthetized by inhaling 3% isoflurane when they were measured cardiac function by ultrasound M‐mode echocardiography.

### 2.8 Echocardiography

The M‐mode echocardiogram system (Vivid i; GE, America) equipped with a 13‐MHz ultrasonic probe (12L‐RS Linear Probe; GE, America) was used to evaluate the cardiac function of rats. The echocardiogram was observed in the left ventricle short axis to measure the left ventricular ejection fraction (LVEF), fractional shortening (FS), left ventricular internal systolic diameter (LVIDs) and left ventricular internal diastolic diameter (LVIDd). Five uninterrupted cardiac cycles were obtained from each rat.

### 2.9 Determination of the biomarkers of myocardial injury

Myocardial injury in vivo was assessed by the activities of AST, LDH, CK and CK‐MB release in plasma using AST, LDH, CK and CK‐MB assay kits, respectively (Nanjing Jiancheng Bioengineering Institute, China). In brief, blood samples were obtained from the abdominal aorta and centrifuged at 2800 *g* for 15 minutes at 4°C to separate the plasma. Approximately 2.0 mL of plasma from each rat was collected. The plasma levels of AST, LDH, CK and CK‐MB were measured according to the protocol of Zhang et al.^18^ Three independent experiments were performed.

### 2.10 Histopathological study

The left ventricles of rats were immersed in 4% paraformaldehyde for 48 hours and then embedded in paraffin as previously described.^19^ Then, 4‐μm‐thick serial sections of left ventricles were obtained. The sections were deparaffinized, rehydrated and stained with Masson's trichrome staining and haematoxylin and eosin (HE) staining. Pathological examination was performed under light microscopy (Axio Imager A2) for observing the structural abnormality.

### 2.11 Terminal deoxynucleotidyl transferase dUTP nick‐end labelling (TUNEL) staining

In Situ cell death detection kit was used to evaluate apoptosis in cardiac tissues. Briefly, the left ventricles of rats were fixed in 4% paraformaldehyde for 36 hours and then embedded in paraffin. Then, 4‐μm‐thick serial sections were made, deparaffinized, dehydrated in graded alcohol and stained by the regents in the In Situ Cell Death Detection Kit. The nuclei stained by haematoxylin were blue, and the apoptotic nuclei stained by Tunel were brownish yellow. All these figures were analysed by Image‐Pro Plus 6.0 (Media Cybernetics).

### 2.12 Western blot analysis

The expression levels of Bax, Caspase‐3, Bcl‐2, PERK, p‐PERK, eIF2α, p‐eIF2α, Chop, GRP78, ATF4, cytochrome C and GAPDH proteins in left ventricular tissues and H9c2 cells were assessed using Western blot. Approximately 100 mg of heart tissues was homogenized by a polytron homogenizer in RIPA buffer with protein phosphatase inhibitor. And H9c2 cells were harvested and digested by the same way. After the lysates were harvested, protein concentrations of samples were measured using a bovine serum albumin kit (Bio‐Rad Laboratories). Analysis of Western blot was carried out as follows: the same amounts of protein were boiled for 10 minutes and separated by SDS‐PAGE on 8%‐12% gels. The proteins were transferred to membranes and then incubated with primary antibodies Bcl‐2 (1:1000), Bax (1:1000), caspase‐3 (1:1000), PERK (1:1000), p‐PERK (1:1000), eIF2α (1:500), p‐eIF2α (1:500), Chop (1:1000), GRP78 (1:1000), ATF4 (1:1000), cytochrome C (1:1000) and GAPDH (1:5000) at 4°C overnight. Then, the membranes were incubated for 2h with secondary antibodies (1:4000) at room temperature. Immunoreactive bands were visualized by using ECL as the HRP substrate. The protein signals of protein bands in membranes were then captured using Image Lab™ Software (Bio‐Rad) and quantified using ImageJ software program.

### 2.13 Statistical analysis

All statistical analyses were calculated by one‐way analysis of variance (ANOVA) followed by Scheffe's post hoc test. Data were obtained from repeated experiments and presented as mean ± SD *P* < .05 was considered to have a statistical difference.

## 3 RESULTS

### 3.1 Fuziline increases cell viability in the model of ISO‐induced injury

**Figure 1:**
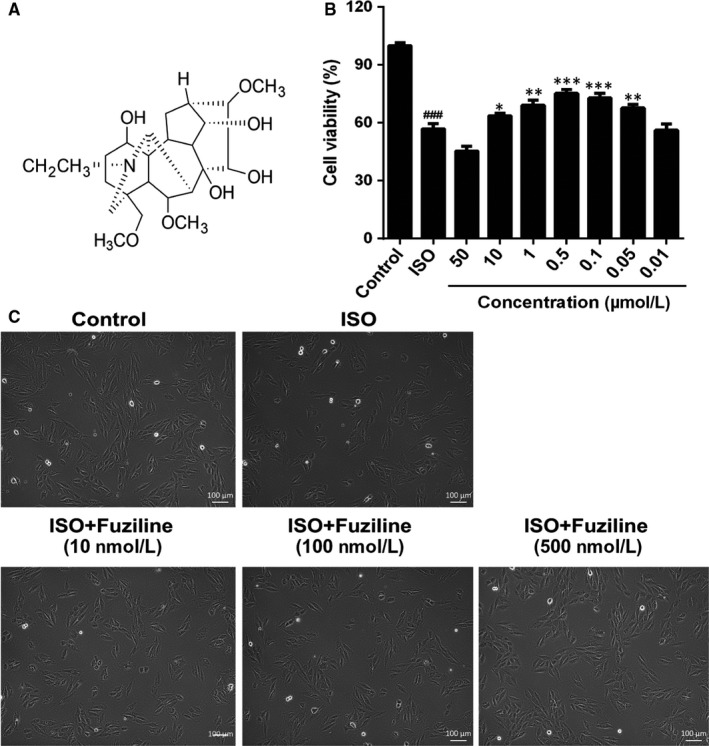
Fuziline increases cell viability in the model of ISO‐induced injury in H9c2 cells. A, The chemical structure of fuziline. B, The effect of fuziline on cell viability was measured by the MTT assay, n = 6. C, The effect of fuziline on the morphology of H9c2 cells (scale bar = 100 μm). ^#^ *P* < .05, ^##^ *P* < .01^, ###^ *P* < .001 vs control group; *P < .05, **P < .01, ***P < .001 vs ISO group. Data are represented as mean ± SD

The structure of fuziline is shown in Figure 1A. H9c2 cells were treated with different concentrations of ISO for 24 or 48 hours, and cell viability was assessed by MTT assay. As shown in Figure S1A, 80 μmol/L ISO for 48 hours significantly decreased cell viability, which was very close to the half inhibitory concentration. Therefore, 80 μM ISO for 48 hours was used in the further experiment. As shown in Figure S1B, a range of concentrations of fuziline were used to evaluate the cytotoxic effect of fuziline on H9c2 cells by MTT assay. While treatment with 100 μmol/L fuziline had a significant inhibition of cell viability, treatment with fuziline (50, 10, 1, 0.5, 0.1, 0.05 and 0.01 μmol/L) for 48 hours had little effect (*P* > .05). Therefore, 50, 10, 1, 0.5, 0.1, 0.05 and 0.01 μmol/L fuziline were selected (Figure 1B). Cell viability in the fuziline (10, 1, 0.5, 0.1 and 0.05 μmol/L) group was higher than the ISO group (*P* < .05). Then, we chose 10, 100 and 500 nmol/L fuziline for the next investigation. Subsequently, the morphology of the cells in different groups was observed under inverted phase‐contrast microscope. As shown in Figure 1C, the morphology of H9c2 cells in ISO group displayed poorly adherent and sparse, while H9c2 cells of 100 and 500 nmol/L fuziline groups became adherent and denser. These results were in line with the MTT assay results, suggesting that fuziline could effectively improve cell survival rate in vitro.

### 3.2 Fuziline alleviates ISO‐induced myocardial apoptosis

**Figure 2:**
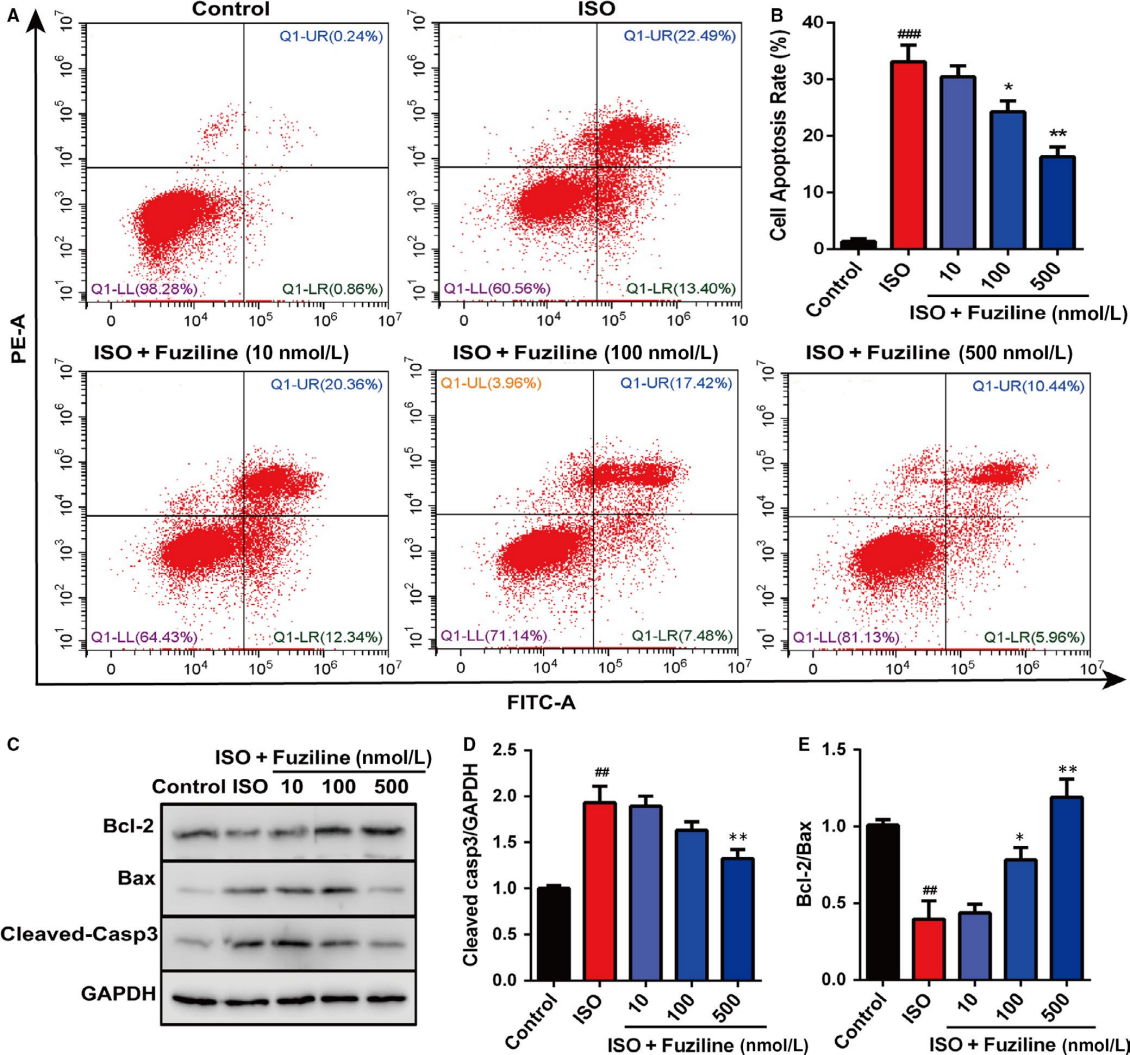
Fuziline inhibits ISO‐induced myocardial apoptosis in vitro. A, The effect of fuziline on cell apoptosis was measured by Annexin‐FITC/PI staining via flow cytometry. B, Quantitative analysis of apoptotic cells in different groups using bar graphs. C, Representative Western blotting bands of Bcl‐2, Bax and cleaved‐caspase 3. D, Quantitative analysis of the ratio of Bcl‐2/Bax by densitometry based on immunoblot images. E, Quantitative analysis of the ratio of cleaved‐caspase 3/GAPDH by densitometry based on immunoblot images. ^#^ *P* < .05, ^##^ *P* < .01, ^###^ *P* < .001 vs control group; *P < .05, **P < .01, ***P < .001 vs ISO group. Data are represented as mean ± SD, n = 3

To determine the ability of fuziline on the inhibition of ISO‐induced myocardial apoptosis, Annexin‐FITC/PI staining analysed by flow cytometry was used. Figure 2A, 2 displays that the ratio of apoptotic cells was obviously decreased at a dose‐dependent manner treated by 100 and 500 nmol/L fuziline compared with the ISO group (*P* < .01). Furthermore, the expressions of apoptotic‐related proteins, including cleaved‐caspase 3, Bcl‐2 and Bax, were detected by Western blotting in H9c2 cells. As shown in Figure 2C, 2, 2, an obvious increase of the ratio of Bcl‐2/Bax and a significant decrease of the ratio of cleaved‐caspase 3/GAPDH were found in 100 and 500 nmol/L fuziline groups by comparison of the ISO group (*P* < .05). All these results clearly suggested that fuziline reduced ISO‐induced myocardial apoptosis to play the cardioprotective role.

### 3.3 Fuziline reduces ISO‐induced oxidative stress injury in H9c2 cells

**Figure 3:**
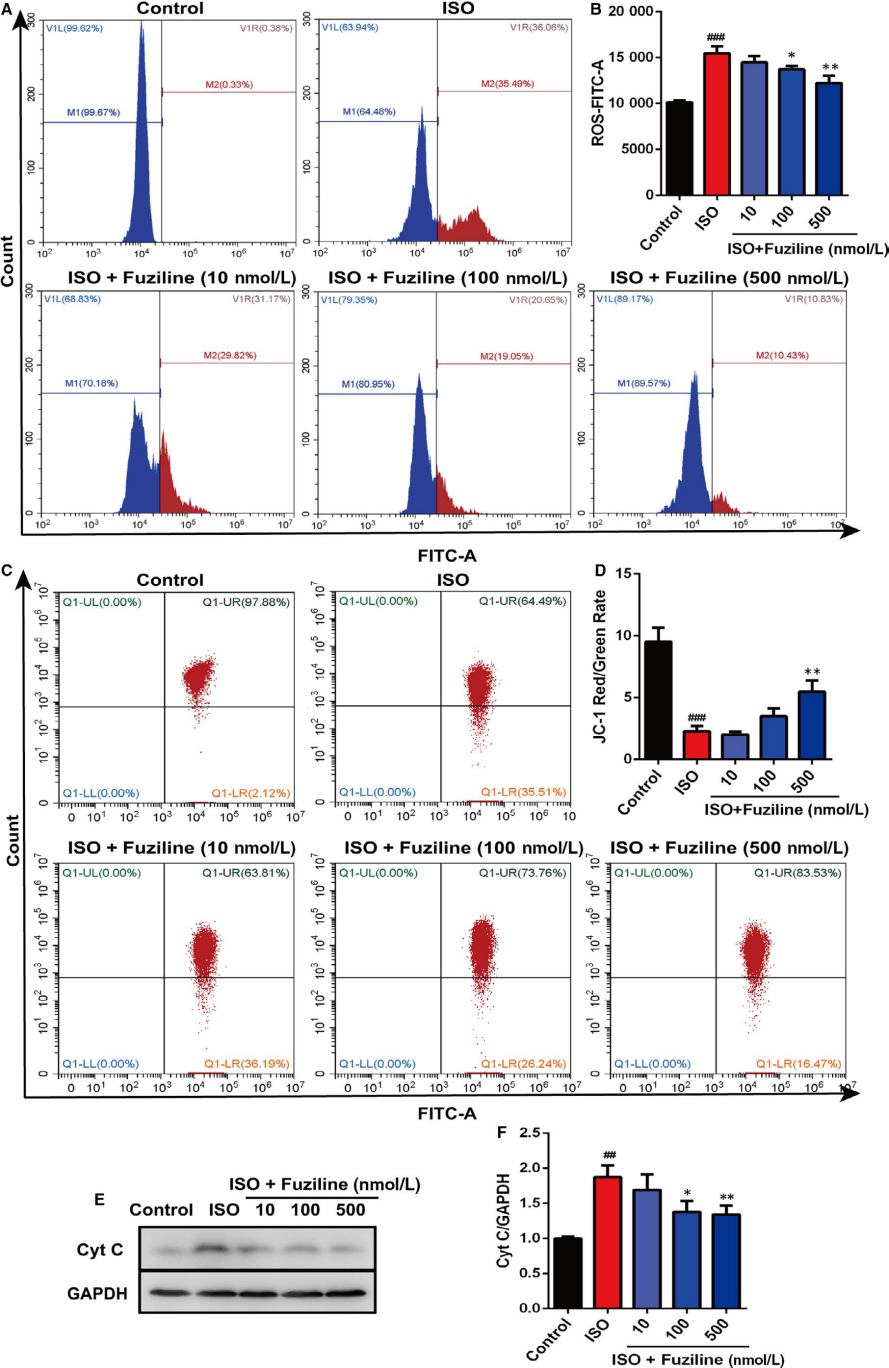
Fuziline reduces ISO‐induced oxidative stress injury in vitro. A, The effect of fuziline on the production of ROS was measured by DCFH‐DA staining via flow cytometry. B, Quantitative analysis of mean DCFH‐DA fluorescence intensity in different groups using bar graphs. C, The effect of fuziline on the changes of MMP was measured by JC‐1 staining via flow cytometry. D, Quantitative analysis of the red‐green fluorescence ratio of the MMP in different groups using bar graphs. E, Representative Western blotting bands of cytochrome C. F, Quantitative analysis of the ratio of cytochrome C/GAPDH by densitometry based on immunoblot images. ^#^ *P* < .05, ^##^ *P* < .01, ^###^ *P* < .001 vs control group; *P < .05, **P < .01, ***P < .001 vs ISO group. Data are represented as mean ± SD, n = 3

In order to explore whether fuziline on the inhibition of apoptosis was related to alleviate oxidative stress injury, DCFH‐DA and MitoSOX Red dye staining were used to evaluate the production of ROS. After treatment with 80 μmol/L ISO for 48 hours, a sharp enhancement of MitoSOX Red fluorescent signals detected by fluorescence microscopy was observed in ISO group, while 100 and 500 nM fuziline treatment reduced MitoSOX Red fluorescent intensity (Figure S2). Flow cytometry was also used to quantitatively measure the changes of ROS production in different groups. As shown in Figure 3A, 3, the intensity of DCFH‐DA in 100 and 500 nM fuziline groups had a sharp decrease compared with ISO group (*P* < .01). Next, we investigated the effect of fuziline on mitochondria. Figure 3C, 3 shows that MMP obviously decreased following ISO treatment reflected by JC‐1 staining, while 100 and 500 nmol/L fuziline treatment dramatically increased the red‐green fluorescence ratio of the MMP with the fluorescent mitochondrial probe JC‐1 (*P* < .01). Then, the expression of mitochondria protein was detected by Western blotting. As shown in Figure 3E, 3, treated by 100 and 500 nmol/L fuziline markedly reduced the release of cytochrome C (*P* < .01). These experimental results suggested that fuziline could effectively inhibit ISO‐induced mitochondria apoptosis and oxidative stress injury.

### 3.4 Fuziline alleviates ISO‐induced endoplasmic reticulum stress injury in vitro

**Figure 4:**
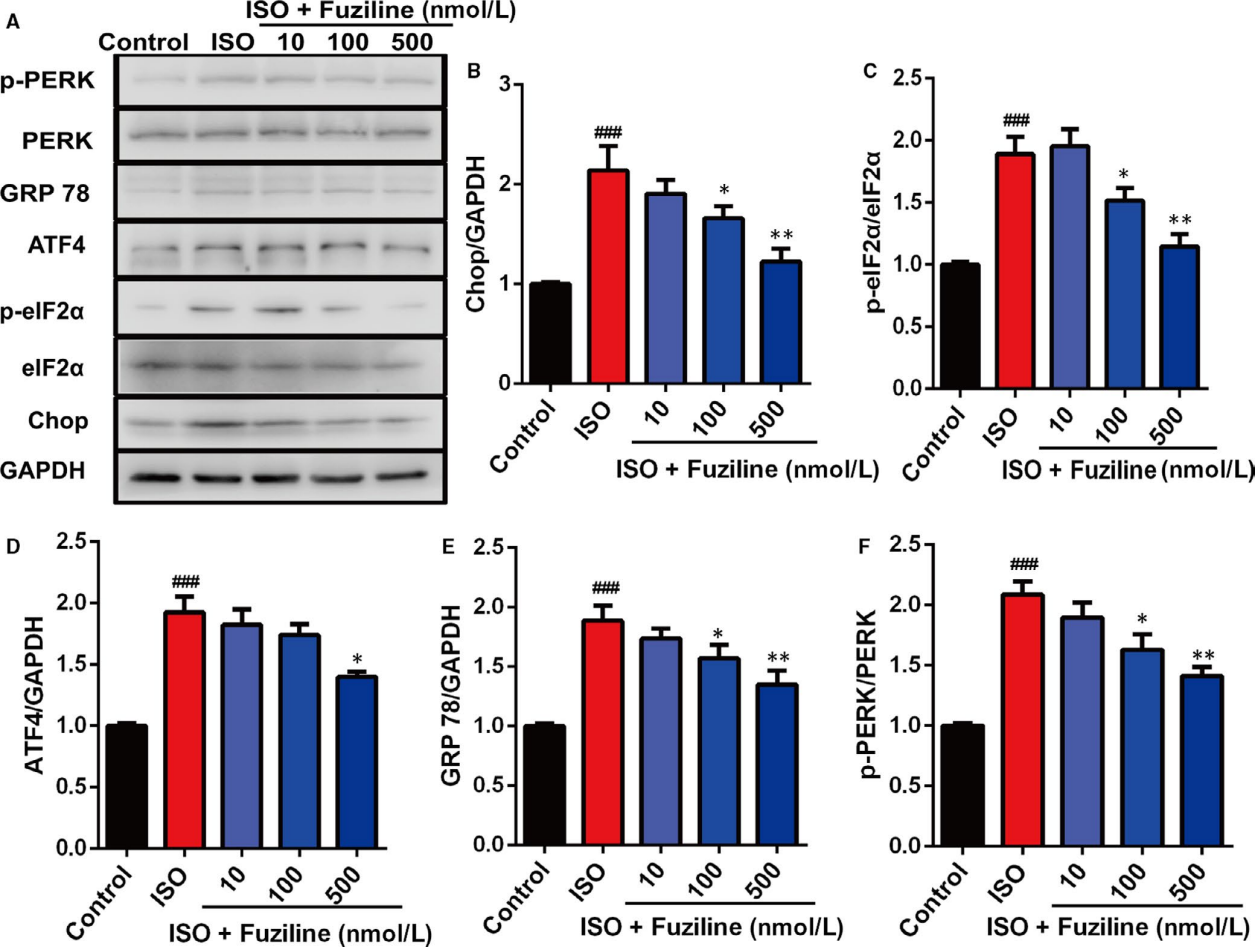
Fuziline inhibits ISO‐induced endoplasmic reticulum stress in H9c2 cells. A, Representative Western blotting bands of ER stress‐related proteins including p‐PERK, PERK, GPR78, p‐eIF2α, eIF2α, ATF4 and Chop. B‐F, Quantitative analysis of the ratios of Chop/GAPDH, p‐eIF2α/eIF2α, ATF4/GAPDH, GPR 78/GAPDH and p‐PERK/PERK by densitometry based on immunoblot images. ^#^ *P* < .05, ^##^ *P* < .01, ^###^ *P* < .001 vs control group; *P < .05, **P < .01, ***P < .001 vs ISO group. Data are represented as mean ± SD, n = 3

The above results clearly indicated that fuziline could reduce ISO‐induced apoptosis and oxidative stress injury in H9c2 cells. A growing number of studies have confirmed that ROS can cause ER stress to induce cell death.^11^ So, we tried to explore whether fuziline on inhibiting apoptosis was related to ER stress. As shown in Figure 4A, 4, the expression levels of ER stress‐related proteins, including Chop, ATF4, p‐eIF2α, eIF2α, GPR78, p‐PERK and PERK, were detected by Western blotting. Treated by 100 and 500 nmol/L fuziline, the ratios of Chop/GAPDH, ATF4/GAPDH, p‐eIF2α/eIF2α, GPR 78/GAPDH and p‐PERK/PERK were significantly down‐regulated compared with the ISO group (Figure 4B), suggesting that fuziline could inhibit ISO‐induced ER stress injury by regulating the PERK/eIF2α/ATF4/Chop pathway.

### 3.5 Inhibition of endoplasmic reticulum stress injury by fuziline is regulated by the production of ROS

**Figure 5:**
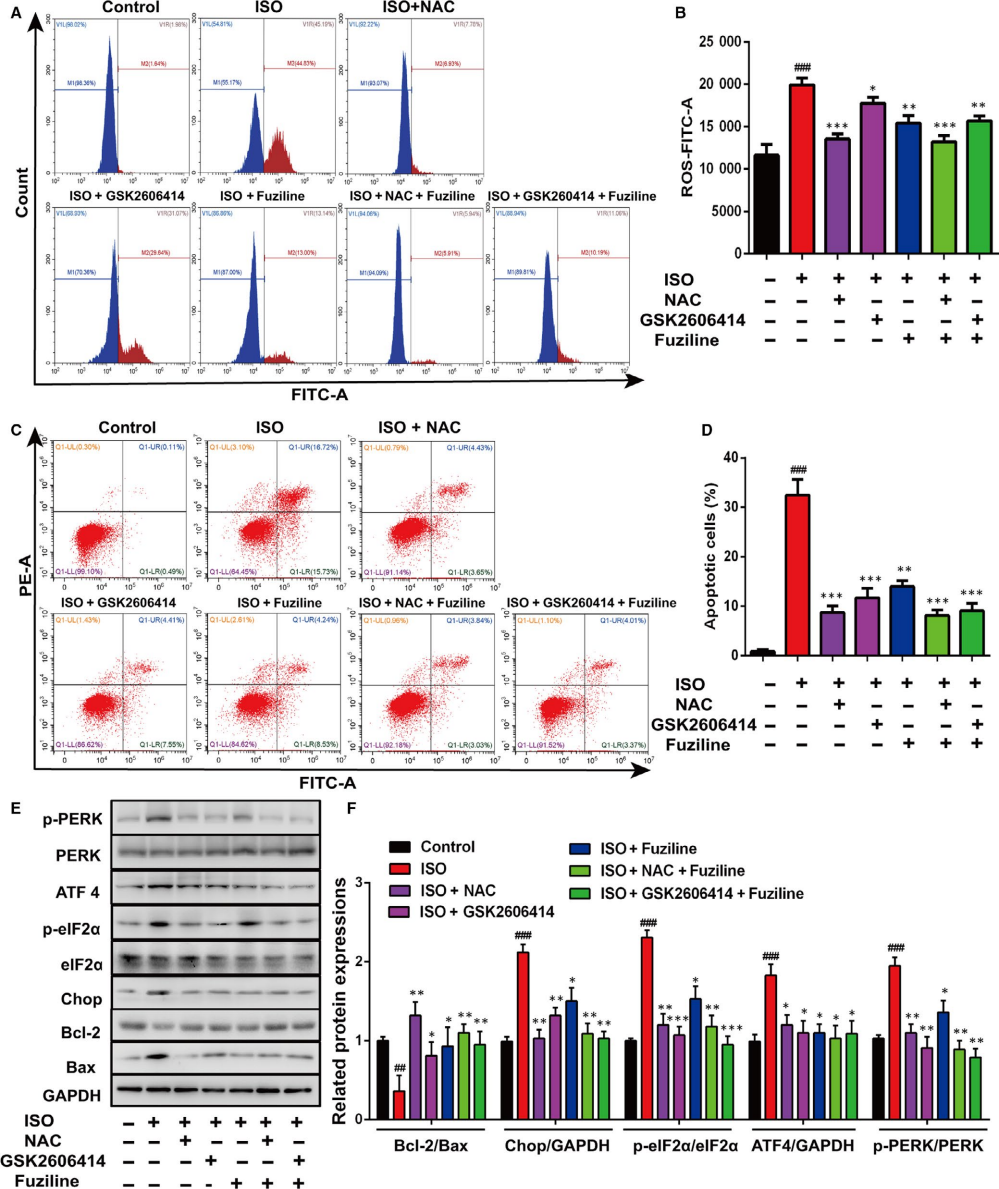
Inhibition of endoplasmic reticulum stress by fuziline is regulated by the production of ROS. A, The effect of NAC, GSK2606414 and fuziline on the production of ROS was measured by DCFH‐DA staining via flow cytometry. B, Quantitative analysis of mean DCFH‐DA fluorescence intensity in different groups using bar graphs. C, The effect of NAC, GSK2606414 and fuziline on cell apoptosis was measured by Annexin‐FITC/PI staining via flow cytometry. D, Quantitative analysis of apoptotic cells in different groups using bar graphs. E, Representative Western blotting bands of apoptosis and ER stress‐related proteins including p‐PERK, PERK, p‐eIF2α, eIF2α, ATF4, Chop, Bcl‐2 and Bax. F, Quantitative analysis of the ratios of p‐PERK/PERK, p‐eIF2α/eIF2α, ATF4/GAPDH, Chop/GAPDH and Bcl‐2/Bax by densitometry based on immunoblot images. ^##^ *P* < .05, ^##^ *P* < .01, ^###^ *P* < .001 vs control group; *P < .05, **P < .01, ***P < .001 vs ISO group. Data are represented as mean ± SD, n = 3

In order to clarify the interplay between ROS and ER stress, the ROS scavenger NAC and the PERK inhibitor GSK2606414 were used. DCFH‐DA staining analysed by flow cytometry demonstrated that NAC (3 mmol/L) and fuziline could significantly reduce the production of ROS, while GSK2606414 (5 μmol/L) did not make a significant decrease on the production of ROS (Figure 5A, 5). However, Annexin‐FITC/PI staining analysed by flow cytometry in Figure 5C, 5 shows that both NAC and GSK2606414 could notably block ISO‐induced apoptosis. These results suggested that ROS triggered ER stress to induce cell apoptosis in the model of ISO‐induced injury in H9c2 cells. To further confirm, related ER stress and apoptotic proteins were detected. Figure 5E, 5 shows that treatment with NAC and GSK2606414 obviously down‐regulated the expressions of UPR‐related proteins ATF4, Chop, p‐PERK, p‐eIF2α and increased the ratio of Bcl‐2/Bax in H9c2 cells. All these results revealed that ROS triggered ER stress injury in the model of ISO‐induced myocardial injury, and fuziline protected against ISO‐induced myocardial apoptosis by inhibiting ROS‐triggered ER stress via the PERK/eIF2α/ATF4/Chop pathway.

### 3.6 Fuziline effectively ameliorates ISO‐induced myocardial injury in vivo

**Figure 6:**
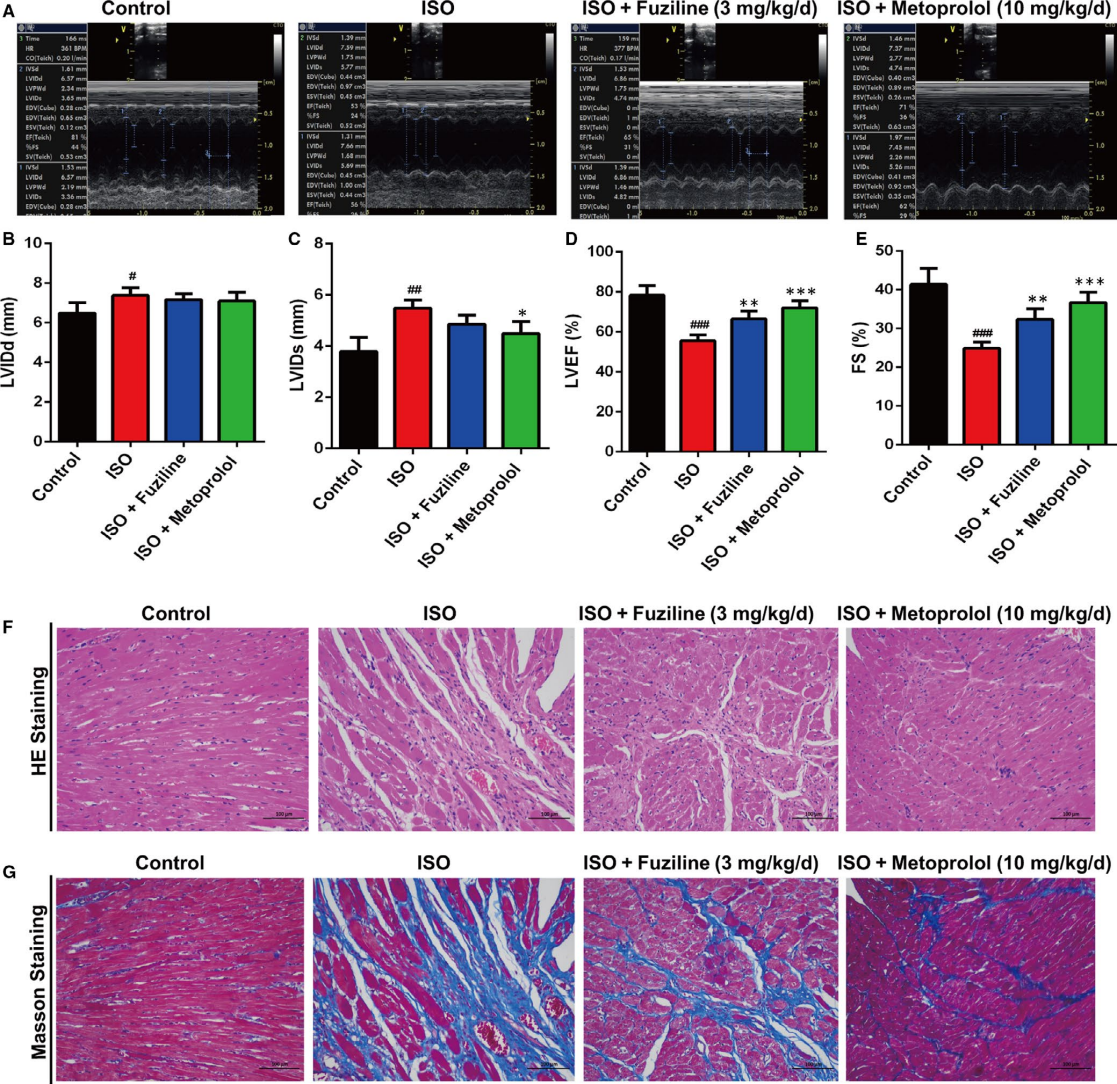
Fuziline treatment ameliorates cardiac function in the rats of myocardial injury induced by ISO. A, Representative M‐mode images of echocardiography for the different groups. B‐E, Quantitative analysis of cardiac function by echocardiography measurement: LVIDd (B), LVIDs (C), LVEF (D), FS (E). F, Representative HE staining images of left ventricles (scale bar = 100 μm). G, Representative images of Masson staining (scale bar = 100 μm). ^#^ *P* < .05, ^##^ *P* < .01, ^###^ *P* < .001 vs control group; *P < .05, **P < .01, ***P < .001 vs ISO group. Data are represented as mean ± SD, n = 3

To evaluate the therapeutic effect of fuziline on ISO‐induced myocardial injury in rats, cardiac indices, such as LVIDd, LVIDs, LVEF and FS, were measured by echocardiography. As shown in Figure 6A, 6, 6, LVEF and FS of the ISO group showed a lower level than the control group, but the LVEF and FS of the fuziline and metoprolol groups were significantly increased (*P* < .01). LVIDd and LVIDs were showed the similar effect, but no significant difference (Figure 6B, 6). In addition, myocardial injury markers, including plasma AST, LDH, CK and CK‐MB, were measured. In accordance with the results of echocardiography, the levels of AST, LDH, CK and CK‐MB in the fuziline and metoprolol groups were lower than those in the ISO group (Figure S3).

In order to assess the protective effect of fuziline on myocardial tissue structure, HE and Masson staining were used. It was apparent from Figure 6F that severe cardiomyocyte necrosis and nuclear dissolution were observed in the ISO group, while myocardial structures were largely maintained in the fuziline and metoprolol groups. Figure 6G directly shows that a bigger area of myocardial fibrosis in the ISO group was observed, while fuziline and metoprolol groups drastically reduced the area of myocardial fibrosis. Moreover, the left ventricular mass index was significantly decreased after treated with fuziline and metoprolol compared with ISO group (*P* < .01, Figure S4A, B). These results clearly suggested that fuziline could improve left ventricular systolic function and alleviate myocardial fibrosis to block ISO‐induced ventricular remodelling.

### 3.7 Fuziline inhibits ISO‐induced myocardial apoptosis by blocking ER stress injury in vivo

**Figure 7:**
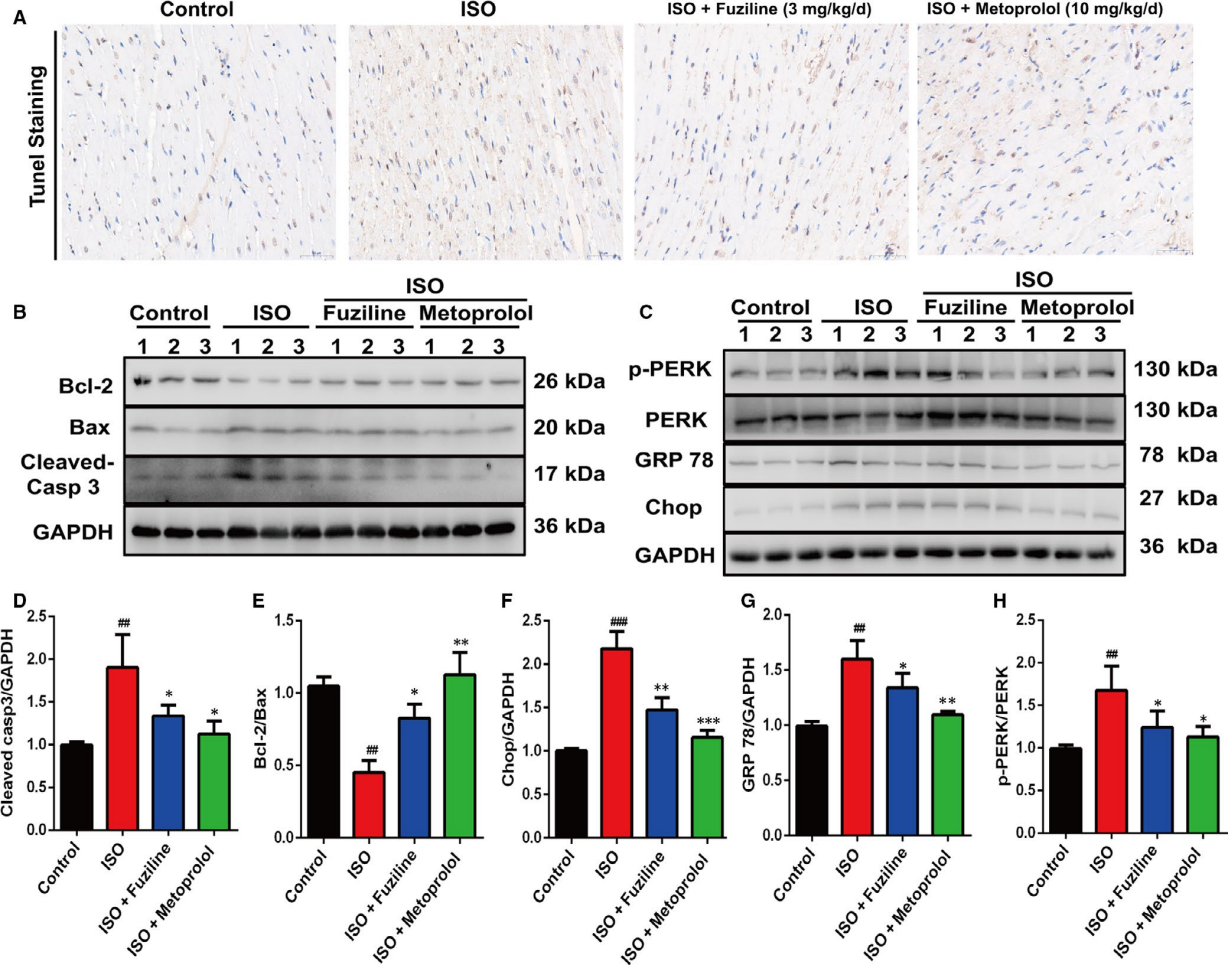
Fuziline inhibits ISO‐induced myocardial apoptosis by blocking ER stress in vivo. A, Representative Tunel staining images of left ventricles for the different groups (scale bar = 100 μm). B, Representative Western blotting bands of apoptosis‐related proteins including Bcl‐2, Bax and cleaved‐caspase 3. C, Representative Western blotting bands of ER stress‐related proteins including p‐PERK, PERK, GPR 78 and Chop. D‐H, Quantitative analysis of the ratios of cleaved‐caspase 3/GAPDH, Bcl‐2/Bax, Chop/GAPDH, GPR 78/GAPDH and p‐PERK/PERK by densitometry based on immunoblot images. ^#^ *P* < .05, ^##^ *P* < .01, ^###^ *P* < .001 vs control group; *P < .05, **P < .01, ***P < .001 vs ISO group. Data are represented as mean ± SD, n = 3

In order to explore the effect of fuziline on ISO‐induced myocardial apoptosis in vivo, we examined apoptosis on cardiac tissues by TUNEL staining. As shown in Figure 7A, the ISO group had more TUNEL‐stained cells than the control group, whereas fuziline and metoprolol treatment notably reduced the numbers of TUNEL‐stained cells. Furthermore, the levels of related apoptotic proteins were measured by Western blotting. The ISO group significantly decreased the ratio of Bcl‐2/Bax and increased the ratio of cleaved‐caspase 3/GAPDH, while fuziline and metoprolol groups showed the opposite effect (*P* < .01, Figure 7B, 7, 7).

ER stress‐related proteins were also measured in cardiac tissues by Western blotting. Figure 7C, 7, G, H shows that the ratios of Chop/GAPDH, GPR78/GAPDH and p‐PERK/PERK in the ISO group were obviously increased compared with the control group, while fuziline and metoprolol treatment decreased these (*P* < .01). Together with the above results, we could find that fuziline inhibited ISO‐induced myocardial apoptosis possibly by blocking ER stress injury in rats.

## 4 DISCUSSION

An increasing number of evidence have confirmed that apoptosis is an important pathophysiological factor leading to cardiac remodelling and heart failure.^20^ Inhibition of apoptosis‐related signal transduction pathways is critical for alleviating myocardial injury.^21^ As we know, mitochondria are not only the centre of cell respiratory chain and oxidative phosphorylation, but also the centre of apoptotic regulation.^22^, ^23^ Mitochondrial‐related apoptosis is usually controlled by the Bcl‐2 family.^24^ According to roles of Bcl‐2 family in apoptosis, they can be divided into two categories: anti‐apoptotic members (eg, Bcl‐2 and Bcl‐xl) and pro‐apoptotic members (eg, Bax and Bak).^25^, ^26^ Among them, anti‐apoptotic proteins, such as Bcl‐2 and Bcl‐xl, mainly exist in mitochondrial membrane, while other members, such as Bid and Bax, mainly exist in cytoplasm.^27^ When cells receive apoptotic‐related signals, Bax will reposition on the surface of mitochondria to form a pore across the mitochondrial membrane, resulting in a decrease of membrane potential and an increase of membrane permeability, thereby mitochondria will release apoptotic factors, such as cytochrome C to induce apoptosis in cells.^28^, ^29^ Studies have showed that the release of cytochrome C from mitochondria was the key step of apoptosis.^30^ Cytochrome C released into the cytoplasm can bind with apoptosis‐related factor‐1 (Apaf‐1) in the presence of dATP to form a polymer and promote caspase‐9 to bind with it to form apoptotic bodies.^31^ Activated caspase‐9 can activate caspase‐3 to induce mitochondrial apoptosis.^31^ In our study, fuziline made an obvious increase of the MMP, up‐regulated the ratio of Bcl‐2/Bax and decreased the release of cytochrome C and the ratio of cleaved‐caspase 3/GAPDH, suggesting that fuziline inhibited ISO‐induced mitochondrial apoptosis in a dose‐dependent manner.

Numerous studies have proved that ROS is a critical factor to induce mitochondrial apoptosis.^11^ ROS plays an important role in many physiological and pathological processes, and excessive production of ROS can destroy the normal function of mitochondria and disrupt normal cell metabolism to induce oxidative stress, ER stress and so on.^11^, ^14^ It has been reported that ISO causes myocardial injury possibly by damaging mitochondrial function via altering mitochondrial membrane permeability and increasing production of ROS.^32^, ^33^ In line with the literature, one of the notable characteristics of ISO‐treated H9c2 cells in our experiment was significant induction of the excess production of ROS. Treated by fuziline effectively reduced the production of ROS in H9c2 cells. In addition, ROS scavenger NAC showed the similar effect to fuziline on the inhibition of cell apoptosis, suggesting that fuziline could alleviate ROS‐induced oxidative stress injury to suppress myocardial apoptosis.

ER is an important site for folding and processing proteins in cells. When synthesis of proteins cannot be folded and transported correctly, a large number of misfolded proteins will accumulate in the endoplasmic reticulum, resulting in ER stress. ER stress is an important mechanism in the pathogenesis of cardiovascular diseases such as hypertensive, myocardial hypertrophy, atherosclerosis, myocardial ischaemia‐reperfusion injury.^34^ Therefore, alteration of the pro‐apoptotic ER stress may be a potential strategy for the treatment of myocardial injury. It has reported that PERK/eIF2α pathway was activated after initiation of ER stress.^35^, ^36^ Chop is considered to be a critical marker of ER stress‐induced apoptosis.^37^ And it is controlled by the PERK‐eIF2α‐ATF4 axis, which promotes the down‐regulation of Bcl‐2 expression resulting in the induction of apoptosis.^37^ In our study, ISO obviously induced ER stress response, which was reflected by elevated levels of Chop, ATF4, p‐eIF2α and p‐PERK, while fuziline inhibited ER stress in H9c2 cells in a dose‐dependent manner. These results in vivo also confirmed that fuziline inhibited ISO‐induced myocardial apoptosis possibly by blocking ER stress. In order to investigate the interplay between ISO‐induced ER stress and apoptosis, GSK2606414 was used to inhibit the function of PERK and block the UPR pathway. And we found that apoptosis was reduced, while the production of ROS was not decreased treated by GSK2606414. Thus, we considered that ISO‐induced apoptosis was downstream of ER stress. To further explore the role of ROS in ISO‐induced ER stress, NAC was applied to decrease the generation of ROS. Our results found that ER stress was alleviated treated by NAC, suggesting that ER stress induced by ISO was partially dependent on the level of ROS. In addition, both blockage of the UPR pathway by GSK2606414 and inhibition of ROS by NAC obviously enhanced the ratio of Bcl‐2/Bax, indicating that ROS‐induced ER stress plays a pivotal role in the ISO‐induced myocardial apoptosis. Using the above evidence, we demonstrated that fuziline protects against ISO‐induced myocardial injury by inhibiting ROS‐triggered ER stress in vitro and in vivo.

## 5 CONCLUSION

This is the first study to investigate the cardioprotective effects of fuziline on ISO‐induced myocardial injury in vitro and in vivo and the potential molecular mechanisms. Our results clearly stated that fuziline protected against ISO‐induced myocardial injury by blocking ROS‐mediated ER stress via PERK/eIF2α/ATF4/Chop pathway. All these results suggested that fuziline may be a potentially therapeutic agent to alleviate myocardial injury. However, more researches such as validation of pharmacodynamics of fuziline in different models of myocardial injury are still needed to be done.

## CONFLICTS OF INTEREST

The authors declare that they have no conflict of interest.

## AUTHORS' CONTRIBUTION

LF, YD and XY designed these experiments. CF and MY were involved in performing the experiment in vitro. CF and GZ were performed the animal experiment and analysed the data. CF and ZY contributed to manuscript preparation and wrote the manuscript. YD, ZY and XY revised the manuscript.

## Data Availability

The data used to support the findings of our study can be obtained from the corresponding authors.
