# Supplementary material for: Fuziline alleviates isoproterenol‐induced myocardial injury by inhibiting ROS‐triggered endoplasmic reticulum stress via PERK/eIF2α/ATF4/Chop pathway
Source: J Cell Mol Med. 2019 Dec 7;24(2):1332–44. doi: 10.1111/jcmm.14803 (PMC6991694; doi:10.1111/jcmm.14803)
Supplement: Supplementary file 1 [file JCMM-24-1332-s001.docx]

**Supplemental Materials**

**Fuziline alleviates** **isoproterenol-induced** **myocardial injury by inhibiting ROS-triggered endoplasmic reticulum stress via PERK/eIF2α/ATF4/Chop pathway**

**Figure S1** Effects of ISO and fuziline on cell death in H9c2 cells.

**Figure S2** Fuziline reduces the production of ROS in mitochondria.

**Figure S3** Fuziline reduces ISO-induced myocardial injury *in vivo*.

**Figure S4** Fuziline alleviates ISO-induced myocardial hypertrophy *in vivo*.





**Figure S1** Effects of ISO and fuziline on cell death in H9c2 cells. A, The viability of H9c2 cells was tested by MTT treated by different concentrations of ISO. B, The effect of different concentrations of fuziline on cell viability in H9c2 cells was measured by the MTT assay. ^#^*p* < 0.05, ^##^*p* < 0.01, ^###^*p* < 0.001, ^*^*p* < 0.05, ^**^*p* < 0.01, ^***^*p* < 0.001. Data represent mean values ± SD, n=6.


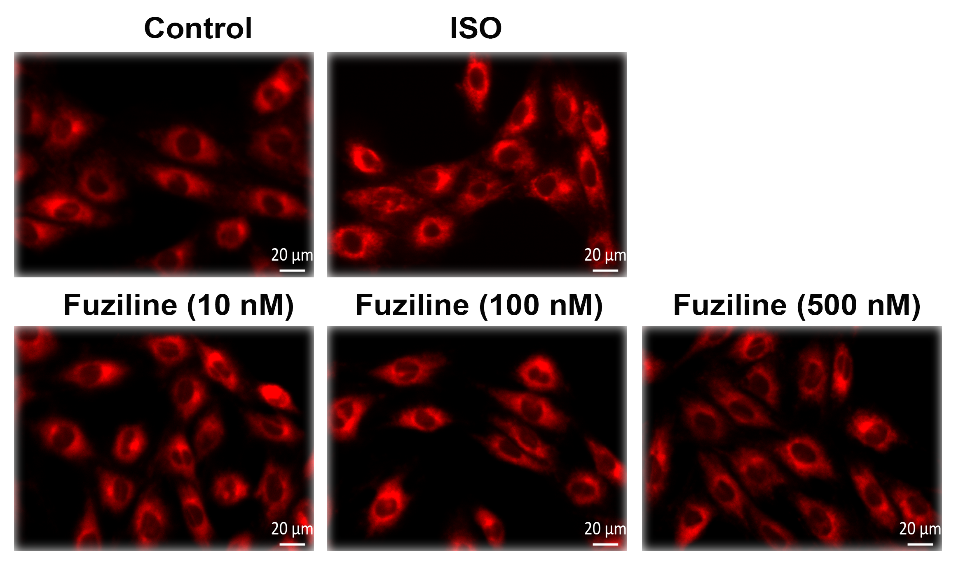


**Figure S2** Fuziline reduces the production of ROS in mitochondria. Representative fluorescent images of MitoSOX Red signals in the H9c2 cells.


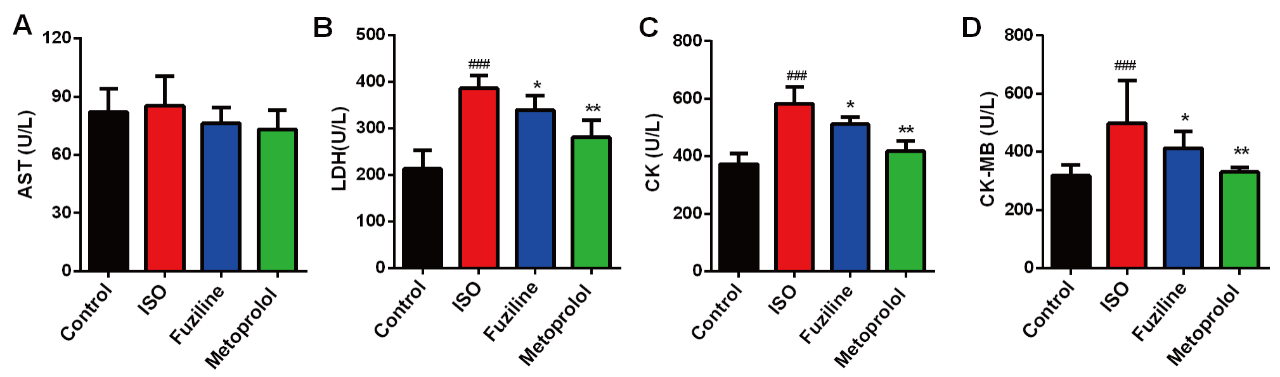


**Figure S3** Fuziline reduces ISO-induced myocardial injury *in vivo*. A-D, Effects of fuziline on the level of myocardial injury markers: plasma AST (A), LDH (B), CK(C) and CK-MB (D). ^#^*p* < 0.05, ^##^*p* < 0.01, ^###^*p* < 0.001 versus control group; ^*^*p* < 0.05, ^**^*p* < 0.01, ^***^*p* < 0.001 versus ISO group. Data represent mean values ± SD, n=6.


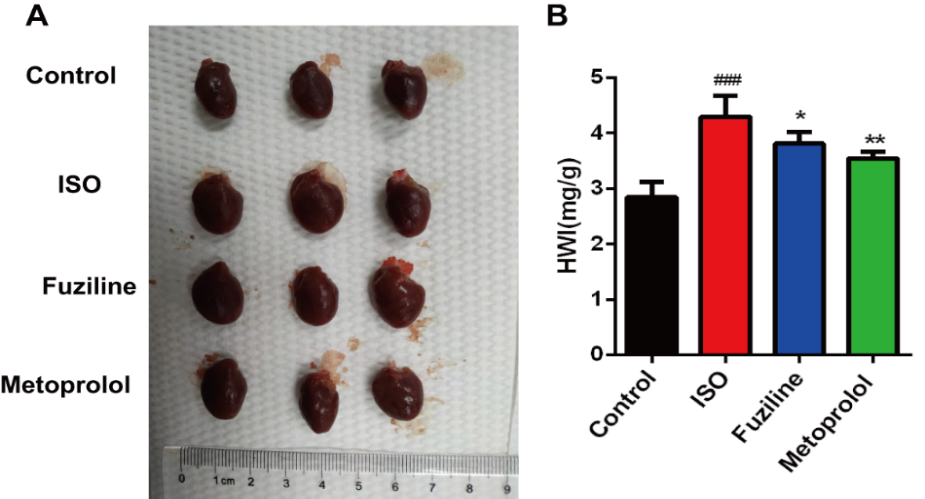


**Figure S4** Fuziline alleviates ISO-induced myocardial hypertrophy *in vivo*. A, Representative fluorescent images of hearts in different groups. B, Effects of fuziline on the level of heart/ weight index (HWI). ^#^*p* < 0.05, ^##^*p* < 0.01, ^###^*p* < 0.001 versus control group; ^*^*p* < 0.05, ^**^*p* < 0.01, ^***^*p* < 0.001 versus ISO group. Data represent mean values ± SD, n=6.
